# Evaluation of an Online Course Promoting Health and Wellbeing for University Students and Employees

**DOI:** 10.3390/ejihpe12090096

**Published:** 2022-09-12

**Authors:** Federico Ricci, Alberto Modenese, Fabriziomaria Gobba, Isabella Morlini

**Affiliations:** 1 Department of Biomedical, Metabolic and Neural Sciences, University of Modena & Reggio Emilia, 41125 Modena, Italy; 2 Department of Economics, University of Modena & Reggio Emilia, 41121 Modena, Italy

**Keywords:** healthy eating, non-sedentary lifestyles, e-learning, knowledge, attitudes, behavior, perceived health, training, healthy lifestyles

## Abstract

Published studies dealing with health promotion activities, such as the improvement of physical activity and healthy eating, for workers and students prove the effectiveness of these preventive interventions. The consequent benefits include better prevention of cardiovascular risk and an improvement in quality of life. Considering this, an intervention aimed at promoting healthy eating and non-sedentary lifestyles has been implemented within an Italian university: the aim of the present research is to evaluate its effectiveness. The intervention consisted of a targeted asynchronous e-learning two-hour course on healthy eating and non-sedentary lifestyles. The attendants were 2004 university students and employees. We conducted two surveys before and after the training intervention, and, through the responses obtained, we evaluated the effectiveness of the intervention. We applied different statistical methods, including unpaired t-tests and nonparametric tests, principal components and cluster analysis. Our results indicate that the post-training knowledge has been significantly improved, compared to that pre-training (7.3 vs. 8.7, *p* < 0.001). Moreover, the whole sample showed an improved awareness of the importance of healthy behaviors, and perception of the University as an institution promoting a healthy lifestyle. Through the principal components analysis, we identified a unidimensional latent factor named “health and behaviors”. The cluster analysis highlighted that the sub-group reporting the lowest scores at the survey before the training was the one with the highest improvement after the intervention. To the best of our knowledge, this is the first Italian study testing, before and after a health promotion intervention, the knowledge and the attitudes and behaviors towards healthy lifestyles of a group of students and workers. Moreover, we also evaluated the pre- and post-intervention perceived health status, as well as the level of engagement of the attendants, with respect to their colleagues and management in an educational institution promoting wellbeing. The conclusions of our study support the need for further adoption of health promotion training interventions, similar to the one we performed, in order to improve healthy eating and non-sedentary behaviors among workers and students.

## 1. Introduction

The COVID-19 pandemic has prompted a radical and rapid change in university life, with particular regard to the massive adoption of remote teaching methods and telework. This can have a relevant impact on the lifestyles of both workers and students, increasing sedentariness and possibly other unhealthy behaviors, including unhealthy nutrition, alcohol consumption and the avoidance of physical activity (PA) [1,2,3,4,5]. Nevertheless, there are also a few exceptions, with reports demonstrating no significant changes in the PA level among university students during the COVID-19 related lockdown [6]. Moreover, there are also concerns on other medium and long-term outcomes affecting the learning process, the self-confidence and the professional skills of the university students, who lacked an adequate face-to-face training [7]. 

Considering the topic of health promoting activities in workplaces, including educational institutions, recently published reviews focused mainly on the need to reduce sedentary behaviors, therefore increasing PA [8,9,10,11,12,13]. The importance of such interventions is sustained by the proved association between an inadequate level of PA and various chronic disorders [14], and is supported by recent World Health Organization (WHO) recommendations [4]. 

The majority of the studies included in the above-mentioned reviews [8,9,10,11,12,13] focused on interventions promoting the use of stairs and of alternative workstations, as well as on traditional exercise-based activities. These interventions seemed effective in increasing PA and reducing sedentary behaviors, while not affecting performance [15]. With particular regard to the pandemic situation and the need for social distancing activities, web technology- and social media-based interventions appear to be promising techniques and effective methods to provide preventive interventions [16]. 

Specific interventions in this field are motivational counselling and trainings, directly involving the participants, with effective results in changing their health behaviors. These types of interventions often also include the use of various technologies, usually connected to the net and with a mobile phone interface [17]. An example of a health promoting activity directly involving college students is that of Pfledderer et al. [18]. This intervention included three different components, i.e., a survey, a 25-min wellness consult with a peer health coach and a 15-min goal planning session, and resulted effective in improving multiple healthy behaviors [18]. Moreover, it should be noted that PA can be practiced at work/school or during leisure time and breaks, i.e., the so called “active rest” [19]. 

Considering now the outcomes adopted to evaluate changes in physical inactivity and sedentary behaviors, many studies measured sitting time, as well as standing and stepping time, with both subjective investigations, as well as with more objective tools, such as specific accelerometers worn by the subjects [3,5,15,17]. 

As indirect indicators of physical inactivity, and excessive sedentary lifestyle, specific questionnaires for the identification of musculoskeletal disorders have been applied, also considering that some evidence suggests that PA interventions may significantly reduce general musculoskeletal pain [20]. Other questionnaires have been applied to evaluate the levels of engagement, satisfaction, performance, stress, wellbeing, general health status and quality of life in workplaces, including educational institutions [8,9,10,11,12,13].

In addition to the interventions related to PA and sedentary lifestyles, many studies include interventions aimed at improving healthy nutrition habits through properly designed collective trainings [21,22]: the joint effect of these two types of intervention proved effective in a recent systematic review [9]. This result is particularly relevant, as studies have shown that various parameters related to the metabolic syndrome, such as waist circumference and triglyceride levels, were significantly higher among subjects with lower PA levels. On the contrary, increased PA prevented factors, such as high LDL or low HDL cholesterol [23,24]. 

The same questionnaires, used to evaluate the health promotion benefits of the interventions to increase PA, are usually also applied also for healthy nutrition interventions, e.g., the previously mentioned tools evaluating individual satisfaction and general health status. Moreover, various studies focus on specific nutrition-related blood parameters, e.g., levels of lipids and glucose, as well as on the anthropometric characteristics of the subjects, such as B.M.I. and neck circumference [9,21,22,23,24,25,26]. Furthermore, in relation to healthy nutrition interventions, other kinds of tests have been applied, with contrasting results, such as specific tests evaluating reaction times to estimate performance, and ad hoc questionnaires measuring subjective mood state [25]. 

Considering the relevant body of published studies on the topic of dietary and PA interventions to promote health at the workplace, including educational institutions, an overall interpretation of the findings is possible. Accordingly, an application of targeted trainings and of short and simple exercises or fitness programs is suggested. Thus, the development and evaluation of interventions to address the specific needs of university students and workers are essential, especially in light of the COVID-19 pandemic, that has significantly modified both workers and students’ lifestyles, increasing sedentary behaviors, reducing PA and favoring unhealthy diets [27].

In this context, an online training course to improve PA, wellness activities and healthy eating has been designed for students and employees of a north-Italian university. To analyze the effectiveness of the course, a test measuring the level of knowledge and education of healthy behaviors was administered before and after the course. Similarly, another questionnaire on the perceived health status, the perceived engagement of the university in promoting wellbeing and the attitudes towards healthy behaviors and the conducts taken up to live a healthy lifestyle within the educational institution, was administered before and after the course. 

The scope of this article is to analyze the data provided, before and after the online course, by the university students and employees who participated in the training. In short, we aim to discuss the benefits of our online course at promoting wellbeing at the university and to evaluate the changes of the students’ and employees’ practices and opinions regarding healthy lifestyles after the intervention.

## 2. Materials and Methods

### 2.1. Study Design and Recruitment

**Figure 1:**
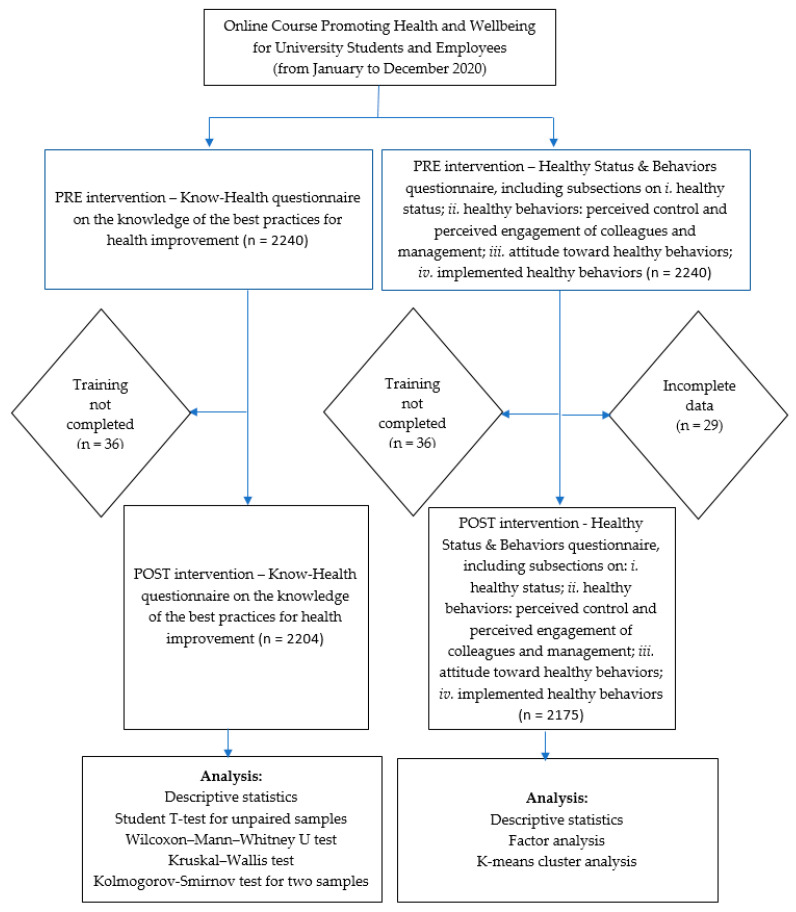
Flow chart describing the design of the study and the analysis performed.

We conducted a study with pre- and post-intervention surveys, based on an electronic mandatory questionnaire administered via the university’s internal platform. Participation in the e-learning course was voluntarily, and took place from January to December 2020. The survey consisted of two different questionnaires, with various sections and subsections, described below. The total target group for the courses included 2240 subjects, both students and employees of the university. The final number of participants who completed the training and filled in the first of the two questionnaires, investigating the knowledge of the best practices for the improvement of the health status (Know-Health questionnaire, see Appendix A), was 2004 subjects. Only 2175 of them properly filled in the second questionnaire (Healthy Status & Behaviors questionnaire, reported in Appendix B, with sections on perceived healthy status; healthy behaviors; perceived control and perceived engagement of colleagues and management; attitude toward healthy behaviors; implemented healthy behaviors). Accordingly, for the remaining 29 subjects, data were incomplete. A flow chart explaining the design of the study and the analysis performed is reported in Figure 1. 

### 2.2. Description of the Intervention

The intervention consisted of a targeted asynchronous e-learning two-hour course, divided into eighteen modules dealing with various aspects of health promotion, including healthy eating and non-sedentary lifestyles. The lectures were registered by specialists in the fields of occupational psychology (F.R.), occupational health (F.G.), exercise science and food and nutrition research [28].

### 2.3. The Measures Considered for the Evaluation of the Training Course 

Following Robson et al. (2012) [29], knowledge, attitudes, behaviors and health perception are the outcomes considered to evaluate the effectiveness of occupational health and safety training. However, we are also interested in considering contextual factors, concerning the perceived engagement of colleagues and of management to implement wellbeing and perceived control of healthy behaviors [30].

The Know-Health questionnaire consists of 10 multiple choices items, randomly selected from a set composed of five sub-sections (see Appendix A): mandatory and not mandatory behaviors, consequences for health of a sedentary life, good practices when using Visual Displays Units (VDU), healthy eating and food properties. For this questionnaire the minimum score achievable was of 0, while the maximum was equal to 10. The same questionnaire was administered before and after the course. 

Similarly, a questionnaire on the self-reported perceptions, named Healthy Status & Behaviors questionnaire (see Appendix B), was administered before and after the course. This survey included an initial question on self-perception and judgement of the healthy status, and then three sub-sections (A, B and C, see Appendix B), as described here below:

- Perceived healthy status: VAS scale of EuroQol-5D (EQ-5D), an instrument for the measurement of quality of life [31].
- Subsection A: Healthy behaviors: perceived control and perceived engagement of colleagues and management. A short (10 item) Italian version of the NOSACQ-50 was adopted for this sub-section, with answers rated on a 7-point Likert scale (from 1 “completely disagree” to 7 “completely agree”) [32].
- Subsection B: Attitude toward healthy behaviors: adapted from Ricci et al. (2018a) [33], with answers based on a 7-point Likert scale, from “absolutely false” to “absolutely true”.
- Subsection C: Implemented healthy behaviors: an ad hoc scale adapted from a checklist, previously applied [34], with answers based on a 7-point Likert scale, from “never” to “ever”.

### 2.4. Staticstical Analysis

The first statistical analysis applied included descriptive statistics of the scores obtained from the surveys and inferential analyses on the means for the Know-Health questionnaire (Figure 1). Then, we performed a factor analysis on the items measured with the Healthy Status & Behaviors questionnaire and a k-means cluster analysis of the respondents (Figure 1). As for this second questionnaire, the respondents were fully anonymized at the source, and it was not possible to compare the contribution of the individual students and employees before and after the course, in this case, the statistical analysis of the effectiveness of the intervention included only a collective evaluation of the data. Moreover, the data cannot be paired, and we considered all the samples as independent. To evaluate the statistical significance of the differences among the knowledge scores registered before and after the course, we first performed the Student’s *t*-test for two unpaired samples, and then we performed non-parametric tests for unpaired samples. According to these tests, the null hypothesis states that the medians of the two populations from which the samples are drown are identical. We opted for the Wilcoxon–Mann–Whitney U two-sample test and for the Kruskal–Wallis test, considering their higher statistical power when applied in large samples, compared to other tests, such as the Mood’s Median Test. These tests are sensitive to differences in scale parameters and symmetry [35]. Finally, we considered the Kolmogorov–Smirnov test for two samples, which compares the two empirical distributions. Considering the Healthy Status & Behaviors questionnaire, to analyze the data obtained before and after the course, we performed a multivariate analysis aimed at identifying the number of latent factors measured by the different items, as well as the coherence of the constructs investigated: healthy behaviors (section A); attitude toward healthy behaviors (section B); and implemented healthy behaviors (section C). Moreover, the analyses were aimed at determining whether the perceived healthy status (000) was correlated with specific items, and for this purpose we applied a principal component analysis of the answers obtained. We constructed biplot graphs in order to appreciate the correlations of the identified components with the perceived healthy status.

Finally, we applied a cluster analysis to classify the attendants based on their answers before and after the course, considering the combinations of all the items. We applied the k-means cluster analysis, a non-hierarchical method, with the Euclidean distance, and we considered the decomposition of the total variance in within and between variances for partitions in 3, 4 and 5 clusters, in order to find the optimal number of groups. The statistical software used for data processing and data analysis is XLSTAT [36].

## 3. Results

### 3.1. Changes in the Knowledge of Courses’ Attendants before and after the Training

**Figure 2:**
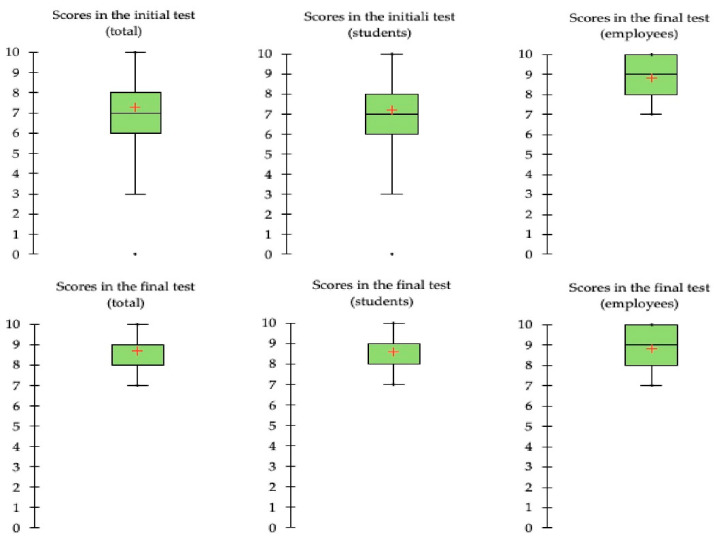
Boxplots of the pre- and post-intervention Know-Health questionnaire scores, obtained in the whole sample and in the two subgroups of students and employees.

The distributions of the scores, graphically represented by boxplots (Figure 2), indicate that the course has improved the level of knowledge on health and wellbeing: the scores at the final test have a lower variability and the scores of the “central” population (i.e., the 50% of the observations closest to the median), represented by the central boxes, increase. We can see that there is a clear increase in the average scores: both the mean and the median increase in the final test, either considering the whole population or the two sub-populations (students and employees). Table 1 reports descriptive statistics concerning pre- and post-intervention Know-Health questionnaire scores, for the whole population and for the subgroups of students and employees.

We then evaluated the differences in the knowledge scores obtained in the two groups before and after the course: the results of the Student’s *t*-test, showing a statistically significant difference both in the whole sample as well as in the subsamples of students and employees (*p* < 0.0001), are reported in Table 2.

Also considering the non-parametric tests for unpaired samples, our results clearly show that, on average, the performances of the attendants, according to the post-intervention scores, are higher than those obtained before the course (Table 3 and Table 4).

**Figure 3:**
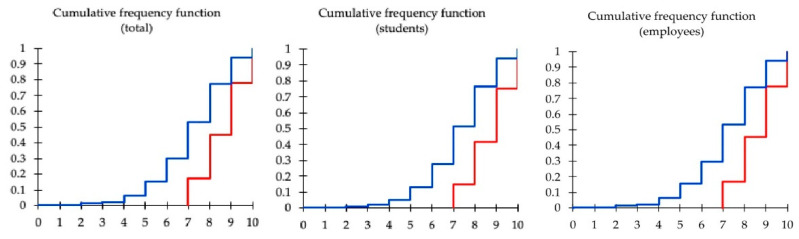
Cumulative frequency functions of the pre- (blue lines) and post-intervention (red lines) Know-Health questionnaire scores obtained by courses’ attendants (students and employees).

Finally, comparing the two empirical distributions, our results prove that the two samples are drawn from different distributions (Table 5) and they have similar shapes (Figure 2 and Figure 3), so we may argue that the samples were different due to the location.

### 3.2. Changes in the Self-Perception of Health and in Healthy Behaviours of Courses’ Attendants before and after the Training

Comparing the means and the medians of the pre- and post-intervention Healthy Status & Behaviors questionnaire scores, we found that, in general, students and employees showed favorable attitudes toward healthy behaviors (Table 6). In particular, the most relevant positive changes after the course involved the sub-section C on implemented healthy behaviors, showing that the attendants improved their habits related to the increase of physical exercise, avoidance of prolonged sitting and of consumption of unhealthy foods after the training (Table 6). Nevertheless, sub-section C was also the one where the lowest means, as well as the highest standard deviations, have been found, indicating a not high frequency of self-reported healthy behaviors, and also a higher variability among respondents. Moreover, for these items, the first quartile is also particularly low, indicating the necessity of promoting healthy lifestyles, particularly in eating and during work (Table 6). 

**Figure 4:**
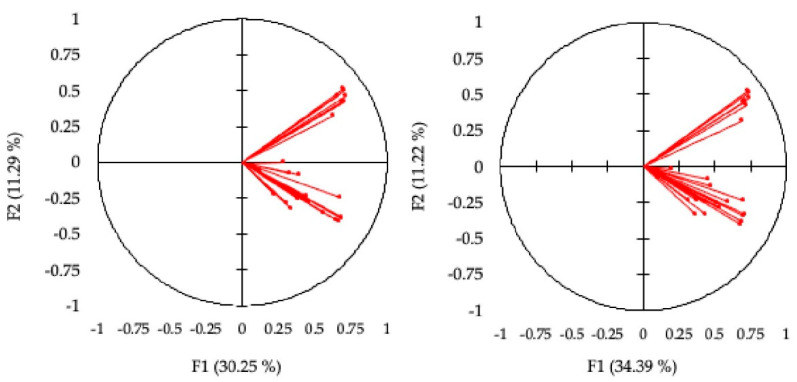
Biplot of the two principal components respective of the pre-intervention (**left side**) and post-intervention (**right side**) Healthy Status & Behaviors questionnaire scores.

The results of the principal components analysis of the answers (standardized) obtained before and after the intervention are very similar. The first component explains the 30.24% of the total variability of the pre-intervention Healthy Status & Behaviors questionnaire scores, and the 35.22% of the total variability of the post-intervention scores. The Cronbach’s alphas are, respectively, 0.88 and 0.91. Both the percentages of variability (higher than the threshold 0.9524 = 0.29) and the values of alpha (close to one) indicate a unidimensional latent factor. Correlations among the variables and the first component are reported in Table 7, as well as the correlations among the variables and the second component (explaining the 11.28% and the 11.22% of the total variability). From the structure of the second component, we can draw some information about the constructs investigated. Figure 4 reports the biplots.

In the unidimensional latent factor analysis, all items are positively correlated with the latent dimension, interpretable as “health and behaviors”. The perceived healthy status (item 000) is moderately correlated with the factor, indicating that how someone feels healthy only moderately influences their opinions and behaviors towards health. The most important items are those related to the perceived engagement of colleagues (04A, 08A, 09A) and of management (05A, 06A, 07A, 10A) in supporting healthy behaviors, and those related to favorable attitudes toward healthy behaviors (items 03B, 04B, 05B, 06B, 07B). The implemented healthy behaviors (section C) are moderately important, indicating the difficulty of translating healthy knowledge and attitudes into practice. This is also confirmed by looking at the questionnaires administered after the course, even though the course was focused on the relevance of behaviors like eating fruits, exercising and limiting the consumption of cured meats. 

Analyzing the biplots and the correlations with the second component, we can see that the perceived healthy status (item 000) is irrelevant for this factor, which is positively correlated only with the items related to the perceived engagement of colleagues and management in supporting healthy behaviors. We may interpret this second factor as the “engagement of colleagues and management of the institution in promoting and implementing health”. This factor is negatively correlated with all the items related to the favorable attitudes toward healthy behaviors (section B) and implemented healthy behaviors (section C). We believe this reveals a second latent factor, which identifies the need for promoting wellbeing and health at the university, even though the workers’ and students’ attitudes toward healthy behaviors are not favorable and they do not implement any particular behaviors for improving health.

**Figure 5:**
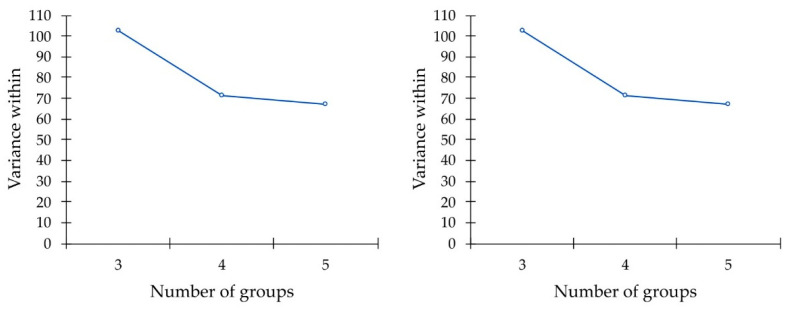
Plot of the within variances of the classifications of the respondents to the Healthy Status & Behaviors questionnaire pre-intervention (**left side**) and post- intervention (**right side**).

Finally, Figure 5 and Table 8 show the results of our cluster analysis, intended to classify respondents before and after having attended the course, considering the combinations of all the items. Figure 5 shows the decrease in the within variance and indicates that four is the optimal number of groups for both the classifications of the respondents before and after the course. 

In order to give insight into the features of respondents belonging to each cluster, Table 8 reports the size of the groups, the cluster’s mean of each item and the *p*-values of the F-test showing the importance of each variable in characterizing the clusters. Groups are ordered considering the mean of perceived health status (item 000). Cluster 1 respondents were those with the highest perceived health status, cluster 4 respondents were those with the lowest perceived health status. 

The profiles of the respondents in each group are quite similar and all items, except for item 02A (i.e., the one examining the interference between healthy behaviors and job activity) and item 06C (i.e., the item related to the habit of eating cured meat less than twice a week), have the same importance in characterizing the groups. Due to the fact that the variables are not standardized, and the distance used is the Euclidean, the perceived health status play a dominant role, as desired (Table 8).

## 4. Discussion

Our study examined the effectiveness of a targeted asynchronous e-learning course proposed by the University of Modena and Reggio Emilia (Italy) for promoting healthy eating and non-sedentary lifestyles among university students and employees. The analysis of the scores obtained from the questionnaires administered before and after the intervention allowed us to investigate the determinants of the attitudes toward healthy behaviors and perceived healthy status. We found that, for both the students and workers who attended the course, the mean values of the pre- vs. post-intervention scores at the questionnaire investigating the knowledge of the subjects on healthy lifestyles were significantly lower for the majority of the items investigated. This indicates that the course had a positive effect, and the effect was particularly clear for those subjects showing a lower self-perception of their health status. This result is in line with the findings of similar technology-based interventions conducted during these times of the COVID-19 pandemic [16].

Considering this, our health promotion course proved effective at increasing the knowledge of the attendants, both students and workers, while for other aspects, such as healthy behavior perception and implementation, some specific observations emerged, as reported here below. 

According to our results, we can note that the level of perceived healthy status influences only some aspects of the favorable attitudes toward healthy behaviors and of the perceived engagement of colleagues and management in promoting wellbeing. In fact, for some items we registered similar mean scores pre- and post-intervention, e.g., for items 03A, 04A, 05A, 03B, 05B and 06B of the Healthy Status & Behaviors questionnaire. On the other hand, for all the items of the section C of the Healthy Status & Behaviors questionnaire (i.e., the section on implemented healthy behaviors), we note that the subjects who reported the lowest scores for these items were also the ones who reported the lowest scores on the healthy status perception scale. After the course, the mean post-intervention scores for all these items have been increased for both workers and students.

Furthermore, analyzing the post-intervention changes within the groups of students and employees with the highest scores on the health status perception scale, we note that the course affects the perceptions of the training attendants. In particular, after the course, the participants increased their feelings that the university management removes obstacles to healthy behaviors (item 10A). They also have more favorable attitudes toward healthy behaviors (items 01B, 02B and 04B) and they are more prone to implement further healthy behaviors (items 03C, 04C, 05C). 

We also note that, in the sample of respondents who attended the course, the mean scores obtained from the questionnaires, stratified according to the level of perceived healthy status, are always equal or higher when compared to the scores obtained by corresponding groups of participants who have not attended the course yet. This indicates that the course has only positive effects, especially for participants with a lower perception of individual health status. Similar data, collected during the COVID-19 pandemic-related lockdown, have been recently reported for another university population: Rivera et al. found that, for the university students who were more sedentary before the lockdown, the health promotion intervention showed the highest values for increasing the PA level [4].

Our results showed that perceived engagement of colleagues and management in promoting wellbeing are important determinants for the students’ and workers’ adoption of healthier behaviors, confirming recent results obtained by Chang et al., who found that health promotion activities were positively associated with peers and supervisors’ support in the workplace [37]. Similarly, for a better promotion of healthy lifestyles among students, it has been shown that the interventions have to be designed taking into account the needs of the recipients. This can lead to an increased motivation for the students, adequately supported by the institution’s management and/or by peer health coaches, to take part in the health promoting initiatives, resulting in increased effectiveness of these activities [18,27]. In particular, we found that the items most correlated with the adoption of positive attitudes towards healthy lifestyles were those related to the perceived engagement of colleagues and of management in supporting healthy behaviors. The cluster analysis showed that the training course affected the perceptions of the investigated subjects, who felt that the management of the institution removed obstacles to the adoption of healthy behaviors, favored positive attitudes towards healthy behaviors and moderately implemented specific healthy behaviors. Moreover, the interference between healthy behaviors and work is not perceived as a constraint in our sample, differently compared to other reports [38,39]. 

The factor analysis showed that the most important unidimensional latent factor, interpretable as “health and behaviors”, was moderately correlated with the self-perception of being in good health conditions. This indicates that how healthy someone feels only moderately influences the opinions and behaviors about health, indirectly confirming that it is risk perception, rather than health perception, that plays a more relevant role in motivating health behavior change [40]. This also happens for the attitudes towards safety procedures at work and can be influenced by factors, such as cultural and ethnic backgrounds [41,42]. Our data also indicates that the efforts of the organization, aimed at implementing healthier behaviors, are only moderately correlated with an actual adoption of healthier practices, supporting published data that shows that it is sometimes difficult to translate knowledge into practice [43]. Nevertheless, it should be noted that our findings cannot be generalized to all the aspects of healthy behaviors, as our pre- vs. post-intervention investigation was mainly focused on the evaluation of specific habits, such as healthy eating (e.g., increase in the consumption of fruits and vegetables, decrease in the assumption of cured meat, etc.) and PA (e.g., performance of simple physical exercises and activities during leisure and working time, increase of non-sedentary resting work-breaks, etc.). We found moderate associations between the implementation of the healthy habits focused on during the health promotion intervention and the adoption of more adequate health practices in our sample. This denotes a further need for promoting wellbeing and health at the workplace, with additional and more effective actions, in line with the objectives of the “Be the Change” program, launched in 2017 by the World Health Organization [44].

Our study has some limitations, and the main is that we were only able to investigate interested subjects, who have attended the health promotion course. Accordingly, the sample is selected and not representative of the entire population. Nevertheless, it should also be noted that the data with the answers to the pre- and post-intervention questionnaires were available for all the subjects who attended the course, with no missing information.

## 5. Conclusions

We collected data before and after an asynchronous e-learning course proposed by the University of Modena and Reggio Emilia (Italy). Our results highlighted, as the most important determinants of more adequate healthy behaviors and habits of the university students and workers, the perception of the engagement and of the support of colleagues and management. This happens when they demonstrate, through their actions, that they consider health and wellbeing promotion of value. The enactment of healthy behaviors is therefore the result of the perceived commitment of colleagues and management in supporting healthy behaviors and favoring attitudes toward healthy behaviors, while not the result of a mere increase in knowledge.

Considering this, future university policies should take into account health promoting actions that encourage healthy behaviors for students and workers. In order to achieve this objective, it is important to support further investigations, within a social system in which the living conditions and wellbeing attitudes of students and employees are fully rewarded. 

## Figures and Tables

**Table 1:** Descriptive statistics of the pre- and post-intervention Know-Health questionnaire scores (based on the number of correct answers to 10 questions, randomly extracted from a pool of questions, 1 point for each correct answer) for the whole population and for the subgroups of students and employees.

	Initial Test			Final Test		
	Total	Students	Employees	Total	Students	Employees
N. of observations	2240	1464	776	2004	1316	688
Minimum	0	0	0	7	7	7
Maximum	10	10	10	10	10	10
1° Quartile	6	6	7	8	8	8
Median	7	7	8	9	9	9
3° Quartile	8	8	8	9	9	10
Mean	7.27	7.20	7.40	8.69	8.60	8.84
Variance	2.70	2.90	2.31	1.02	1.02	0.958
Standard deviation	1.64	1.70	1.52	1.01	1.01	0.980
Coefficient of variation	0.23	0.24	0.21	0.12	0.12	0.11
Skewness (Pearson)	−0.84	−0.79	−0.90	−0.21	−0.12	−0.37
Curtosys (Pearson)	1.49	1.25	1.99	−1.05	−1.08	−0.90

**Table 2:** Results of the Student’s *t*-test for two unpaired samples to evaluate differences in the pre- and post-intervention Know-Health questionnaire scores obtained by the subjects.

	Total	Students	Employees
Mean difference	−1.410	−1.399	−1.434
*t* (observed)	−34.093	−26.650	−21.695
*p*-value (one tail)	<0.0001	<0.0001	<0.0001

**Table 3:** Results of the Wilcoxon–Mann–Whitney U two-samples test. Descriptive statistics of the pre- and post-intervention Know-Health questionnaire scores obtained by courses’ attendants (students + employees).

	Total	Students	Employees
U	1,086,335	486,044	117,156
U (normalized)	−29.731	−23.094	−19.003
Expected value	2,244,480	963,312	266,944
Variance (U)	1,517,451,164	427,096,011	62,127,400
*p*-value (one-tailed)	<0.0001	<0.0001	<0.0001

**Table 4:** Results of the Kruskal–Wallis two-samples test (one tail) applied to the pre- and post-intervention Know-Health questionnaire scores obtained by courses’ attendants (students + employees).

	Total	Students	Employees
K (observed)	883.916	482.605	354.168
Degrees of freedom	1	1	1
*p*-value (one-tailed)	<0.0001	<0.0001	<0.0001

**Table 5:** Results of the Kolmogorov–Smirnov two-samples test applied to the pre- and post-intervention Know-Health questionnaire scores obtained by courses’ attendants (students + employees).

	Total	Students	Employees
D	0.368	0.360	0.407
*p*-value (one-tailed)	<0.0001	<0.0001	<0.0001

**Table 6:** Descriptive statistics of the pre- and post-intervention scores obtained by courses’ attendants (students and employees) at the Healthy Status & Behaviors questionnaire.

		Respondents Pre-Intervention (n = 2240)					Respondents Post-Intervention (n = 2175)				
Subsection	Item Number/Question	1st Quartile	Median	3rd Quartile	Mean	Stand. Dev.	1st Quartile	Median	3rd Quartile	Mean	Stand Dev.
Perceived health status *	000 How do you judge your health status in this moment? *	75	85	90	81.7	16.6	70	85	90	80.1	19.1
Healthy behaviors: perceived control and perceived engagement of colleagues and management **	01A I think it is important to implement healthy behaviors	7	7	7	6.8	0.6	7	7	7	6.8	0.6
	02A Healthy behaviours do not interfere with my job activity	2	5	7	4.5	2.2	3	6	7	4.9	2.2
	03A I spontaneously implement healthy behaviours, without somebody’s help	6	6	7	6.1	1.1	6	6	7	6.1	1.0
	04A Workers in this institution think that education courses in healthy behaviors are useful	6	7	7	6.1	1.1	6	7	7	6.3	1.1
	05A The management of this institution works in order to inform about healthy behaviors	5	6	7	6.0	1.2	6	6	7	6.2	1.1
	06A The management of this institution constantly supports healthy behaviors	5	6	7	5.7	1.4	5	6	7	5.9	1.3
	07A The management of this institution involves workers in decisions about healthy behaviors	5	6	7	5.4	1.5	5	6	7	5.6	1.5
	08A Workers in this institution help each other in adopting healthy behaviors	4	6	6	5.3	1.5	5	6	7	5.5	1.5
	09A Workers in this institution discuss adopting healthy behaviors	5	6	7	5.6	1.4	5	6	7	5.8	1.3
	10A The management of this institution removes obstacles to healthy behaviors	4	6	6	5.3	1.5	5	6	7	5.6	1.4
Attitude toward healthy behaviors ***	01B If I implement healthy behaviors, then I prevent health problems	6	7	7	6.3	1.0	6	7	7	6.5	0.8
	02B If I don’t implement healthy behaviors, I feel guilty	5	5	6	5.2	1.5	5	6	7	5.6	1.4
	03B Implementing healthy behaviors reassures myself	5	6	7	6.0	1.2	6	6	7	6.1	1.1
	04B When I implement healthy behaviors, I feel better	6	7	7	6.3	1.0	6	7	7	6.4	0.9
	05B Implementing healthy behaviors calms myself	5	6	7	6.0	1.2	6	6	7	6.1	1.1
	06B I think I will implement healthy behaviors in the next weeks	6	6	7	6.2	1.1	6	7	7	6.3	0.9
	07B In the next weeks I will encourage healthy behaviors	5	6	7	5.8	1.3	6	6	7	6.1	1.1
Implemented healthy behaviors ****	01C I exercise for at least 150 min a week	3	5	7	4.8	1.9	4	6	7	5.2	1.8
	02C Working at VDU, I take a break every two hours to rest my eyes	3	5	6	4.4	1.9	4	5	6	5.1	1.7
	03C Every day, I eat at least 400 g of fruits and vegetables	3	4	6	4.3	1.9	4	5	6	4.9	1.8
	04C During work, I stand up to interrupt the inactive position	4	6	7	5.2	1.6	5	6	7	5.6	1.4
	05C I drink at least 2 liters of water every day	4	5	7	4.9	1.8	4	6	7	5.3	1.7
	06C I eat cured meat less than two times a week	3	5	7	4.8	2.0	4	5	7	5.0	1.9

* items’ scores based on a visual analogic scale from 0 to 100; ** scores based on a 7-point Likert scale, from 1 “completely disagree” to 7 “completely agree”; *** scores based on a 7-point Likert scale, from “absolutely false” to “absolutely true”; **** scores based on a on 7-point Likert scale, from “never” to “ever”.

**Table 7:** Correlations among the pre- and post- intervention Healthy Status & Behaviors questionnaire scores and the first and the second principal components. In bold, positive correlation of items regarding perceived engagement of colleagues and management of the institution in promoting and implementing health, with the second component.

	Pre-Intervention Scores		Post-Intervention Scores	
Item	First Component	Second Component	First Component	Second Component
000	0.291	0.000	0.199	−0.015
01A	0.442	−0.234	0.512	−0.258
02A	0.124	−0.057	0.101	−0.080
03A	0.468	−0.276	0.531	−0.284
04A	0.633	**0.322**	0.691	**0.313**
05A	0.662	**0.463**	0.703	**0.445**
06A	0.711	**0.498**	0.745	**0.518**
07A	0.704	**0.514**	0.735	**0.522**
08A	0.717	**0.468**	0.747	**0.476**
09A	0.709	**0.422**	0.724	**0.425**
10A	0.688	**0.424**	0.716	**0.453**
01B	0.390	−0.257	0.435	−0.248
02B	0.563	−0.350	0.596	−0.249
03B	0.695	−0.395	0.708	−0.346
04B	0.656	−0.403	0.697	−0.381
05B	0.695	−0.381	0.709	−0.331
06B	0.667	−0.409	0.683	−0.406
07B	0.685	−0.247	0.700	−0.238
01C	0.306	−0.288	0.373	−0.339
02C	0.329	−0.076	0.456	−0.087
03C	0.343	−0.322	0.440	−0.338
04C	0.400	−0.088	0.477	−0.138
05C	0.325	−0.187	0.373	−0.237
06C	0.224	−0.228	0.321	−0.240

**Table 8:** Results of the cluster analyses of the Healthy Status & Behaviors questionnaire administered pre- and post-intervention.

	Pre-Intervention						Post-Intervention					
Item	F	*p*-Value	Group 1	Group 2	Group 3	Group 4	F	*p*-Value	Group 1	Group 2	Group 3	Group 4
			n = 1067 (47%)	n = 696 (31%)	n = 432 (19%)	n = 69 (3%)			n = 900 (46%)	n = 551 (28%)	n = 426 (22%)	n = 98 (5%)
000	6618	0.000	94	80	65	18	7202	0.000	94	81	66	15
01A	17.9	0.000	7	7	7	6	10.2	0.000	7	7	7	7
02A	2.4	0.064	5	5	4	4	1.9	0.131	5	5	5	5
03A	23.6	0.000	6	6	6	6	39.5	0.000	6	6	6	6
04A	20.2	0.000	6	6	6	6	15.2	0.000	6	6	6	6
05A	13.4	0.000	6	6	6	6	15.1	0.000	6	6	6	6
06A	13.8	0.000	6	6	6	5	22.0	0.000	6	6	5	6
07A	12.8	0.000	6	5	5	5	17.1	0.000	6	6	5	6
08A	14.9	0.000	6	5	5	5	14.8	0.000	6	5	5	5
09A	19.1	0.000	6	6	5	5	17.8	0.000	6	6	6	5
10A	18.5	0.000	5	5	5	5	17.6	0.000	6	6	5	5
01B	10.3	0.000	6	6	6	6	5.0	0.002	7	7	6	6
02B	10.3	0.000	5	5	5	5	12.9	0.000	6	5	5	5
03B	19.2	0.000	6	6	6	6	9.1	0.000	6	6	6	6
04B	14.3	0.000	6	6	6	6	9.1	0.000	7	6	6	6
05B	15.2	0.000	6	6	6	6	10.6	0.000	6	6	6	6
06B	22.0	0.000	6	6	6	6	13.5	0.000	6	6	6	6
07B	14.7	0.000	6	6	6	5	15.6	0.000	6	6	6	6
01C	12.8	0.000	5	5	4	5	18.0	0.000	5	5	5	5
02C	10.0	0.000	5	4	4	4	13.2	0.000	5	5	5	5
03C	7.8	0.000	4	4	4	3	10.9	0.000	5	5	4	5
04C	21.2	0.000	5	5	5	4	17.8	0.000	6	6	5	5
05C	10.4	0.000	5	5	5	4	13.0	0.000	6	5	5	5
06C	2.6	0.051	5	5	5	4	5.4	0.001	5	5	5	5

## Data Availability

The data presented in this study are available on request from the corresponding author. The data are not publicly available due to privacy reasons.

## Appendix A. The Know-Health Questionnaire on the Knowledge Related to Healthy Lifestyles (In Bold the Correct Answer)

**Section 1**
1. Our behavior is mainly the consequence of: - **habits** - obliged choices - casual events
2. Every one of us can be more active, from a physical point of view: - no - **yes** - sometimes
3. By repeating behaviors that increase our physical activity, we can develop a lifestyle: - more sedentary - more regular - **healthier**
4. The employer must give to employees a proprioceptive pillow and a fitness ball: - yes, but only one of them - yes, to both of them - **no**
5. The use of the proprioceptive pillow and of the fitness ball at work, when not in contrast with the role and work activity, is: - **a personal choice** - a mandatory behavior based on legislation - a recommended behavior based on internal procedures
**Section 2**
6. Which tumors have proved associations with sedentary work? - **breast and colon cancers** - results are incoherent, and accordingly, at the moment, it is not possible to conclude for any association - with some tumors, but only for diabetic subjects - there is no scientific demonstration of any association between sedentary work and any type of tumor
7. Can sedentary work be associated with blood hypertension? - **yes** - results are incoherent, and accordingly, at the moment, it is not possible to conclude for an association - yes, but only for obese or overweight subjects - there is no scientific demonstration of any association between sedentary work and hypertension
8. Physical activity can lead to better health: - this is true, but specifically for healthy individuals - this is true, but specifically for overweight subjects - **this is true, for all the population** - no, this is not true
9. An increase in physical activity can lead to better health outcomes, with specific regard to diseases such as: - diabetes and vein thrombosis, while not for hypertension - hypertension and diabetes, while not for vein thrombosis - vein thrombosis and hypertension, while not for diabetes - **diabetes, hypertension and vein thrombosis**
10. Prolonged sitting time, compared to adequate walking and going up/down stairs time, showed an association with cardiovascular mortality: - **higher** - lower - equal - results are incoherent, and accordingly, at the moment, it is not possible to conclude for any association
11. Any interruption of prolonged sitting at work can reduce the risks for health: - false, this happens only in 3 out of 4 cases - **true** - this is true only for those sat for more than 11 h per day - true, only in the 40% of the examined cases
12. Workers who are less sedentary compared to others: - experience more injuries - **are more productive** - have lower exposures to risks for occupational diseases - don’t show particular differences
13. According to the WHO, in order to achieve a better health status, it is necessary to perform physical activity for at least: - 50 min/week - **150 min/week** - 50 min/day - 150 min/day
14. The promotion of physical activity at work should include interventions: - on work organization, with no focus on an individual self-evaluation of healthy behaviors with specific initiatives aimed at encouraging workers - **aimed at a self-evaluation of healthy behaviors with specific initiatives to favor and encourage the correct behaviors for workers** - aimed at a self-evaluation of healthy behaviors, but without specific interventions to favor and encourage the correct behaviors for workers
15. Which one, among the following, cannot be considered a useful proposal to reduce sedentariness at work? - **indication to avoid in-person exchanges of words with colleagues, as it is possible to use internal email/chat services** - indication to use a printing machine located at distance from the office - indication to increase the intake of drinking water, in order to increase the need for more breaks to go to the toilet - indication to go to the workplace by foot or bike, or, if not possible, at least to park the car/motorbike far from the entrance of the building
16. In order to improve physical activity at work, which one is the incorrect action: - a gradual introduction of the preventive measures - the choice of activities that can be enjoyable for the workers - an input-based program (quantity of activities) - **focusing on the final outcome (e.g., quantification of the loss of weight)**
**Section 3**
17. During work at the Visual Display Unit, the direction of the gaze of the worker should be a bit inclined downwards and the monitor has to be placed: - 10–15 cm distant from the worker - **in front of the worker** - beside the worker - in front of the window
18. During work at the Visual Display Unit, the keyboard has to be placed: - **10–15 cm distant from the border of the desk** - at a 90-degree angle to the screen - always in front of the window - as close as possible to the screen
19. Considering the work at the Visual Display Unit, which one among the following is the incorrect affirmation? - the keyboard has to be in front of the monitor - the worker needs to stay 50–70 cm distant from the monitor - **no health surveillance has to be warranted to workers operating Visual Display Units** - the screen has to be placed beside the window, if possible
20. Considering the work at the Visual Display Unit, the chair has to be adapted by: - varying the height of the seat and also modifying the inclination of the backrest, but without altering the height of the backrest - changing the height and inclination of the backrest, while maintaining the seat height unchanged - changing the height of the backrest and seat, but without affecting the inclination of the backrest - **varying the height of the seat and of the backrest, and modifying the inclination of the backrest**
21. Considering the work at the Visual Display Unit, which one among the following is the incorrect affirmation? - the worker’s feet must be well placed on the ground - the worker’s knees must form a 90 ° angle - **the direction of the operator’s gaze must be upwards** - the worker must take a break every 2 h, resting his gaze
22. Breaks in case of Visual Display Unit work are required: - only in case of specific diseases affecting the worker, certified by an occupational physician - **to have a rest of the eyes for 15 min every 2 h** - for a total amount of 2 h per week - to look away from the screen, but without leaving the workstation
**Section 4**
23. Which of the following foods is at the base of the Food Pyramid, considering the adult Mediterranean diet? - **cereals and derivatives** - red meat - milk - nuts and spices
24. At the base of the Food Pyramid of the Mediterranean diet there are the foods that should be eaten: - occasionally - **frequently** - rarely - very rarely
25. The Food Pyramid is aimed at: - transmitting to posterity knowledge about ancient foods that are no longer in use - facilitating the knowledge of food uses in ancient Egypt - **giving advice on the adequate quantities of different food intake** - enhancing the food and wine excellence of a territory
26. The word diet originates from ancient Greek and can be translated to the term: - food - **lifestyle** - physical activity - well-being
27. It is advisable to alternate fruit and vegetables according to the 5 colors because a different color is also associated with a different: - degree of aesthetic gratification - economic value - **phytonutrient content** - way of consuming food
28. How many times a day is it recommended to eat fruit and vegetables? - 2 - **5** - 3 - 6
29. How many times a week, at least, is it recommended to eat legumes? - more than 10 - **2–3** - on a daily basis - about 10
30. The WHO recommends consuming fruit and vegetables every day, of at least: - 500 g - 900 g - **400 g** - 200 g
31. Where can we not find protein? - meat and fish - milk and cheese - **vegetables** - legumes
32. Dietary intake should be particularly limited of: - olive oil - herbs and spices - **animal fats** - seed oils
33. Which of the following foods is it advisable to consume more frequently? - cured meat - sweets - **pasta** - cheeses
34. What is the maximum daily intake of table salt recommended by the World Health Organization for healthy individuals? - 1 g - 3 g - **5 g** - 10 g
35. What is the maximum daily intake of added sugars, recommended by the World Health Organization, in relation to the total energy intake of an individual? - **10%** - 1% - 3% - 15%
36. Which of the following foods does not have a high nutritional content? - spaghetti with tomato sauce - vegetable salad with tuna - apple - **slice of “cheesecake”**
37. What is the daily dietary fiber intake recommendation? - 50 g - 5 g - **25 g** - 35 g
38. 150 g of wholemeal pasta contains 10 g of fiber; the same amount of fiber is contained in how many grams of regular pasta? - **300 g** - 200 g - 250 g - 500 g
39. Assuming a daily intake of 2000 Kcal, how many of them should be, in proportion, for breakfast? - **400** - 200 - 300 - 500
40. Assuming a daily intake of 2000 Kcal, how many of them should be, in proportion, for lunch? - **600** - 300 - 400 - 800
41. Assuming a daily intake of 2000 Kcal, how many of them should be, in proportion, for a snack? - **200** - 100 - 300 - 400
42. The “Calorie density” is the quantity of: - added sugars - fats, by percentage of food weight - time to cook or transform the food - **calories per gram**
43. The “Calorie density” of the celery is: - high - undefined - it depends on the cultivation process - **low**
**Section 5**
44. The body mass index must be: - <18.5 - >25 - **18.5–25**
45. Over half of the calories we need should come from: - **carbohydrates** - meat and fish - milk and cheese
46. Over a third of the calories we need should not come from: - **fats** - pasta and rice - legumes
47. The recommended daily intake of drinking water is at least: - 5 L - **2 L** - 3.5 L

## Appendix B. The Healthy Status & Behaviors Questionnaire

Items concerning perceived healthy status (000), perceived control of healthy behaviors (01A, 02A, 03A), perceived engagement of colleagues (04A, 08A, 09A) and of management (05A, 06A, 07A, 10A) to implement wellbeing (section A), attitude toward healthy behaviors (section B), implemented healthy behaviors (section C).

**Label**	**Question**
**000**	How do you judge your healthy status in this moment?
**Section A**	
**Healthy behaviors: perceived control and perceived engagement of colleagues and management**	
**01A**	I think it is important to implement healthy behaviors
**02A**	Healthy behaviors do not interfere with my job activity
**03A**	I spontaneously implement healthy behaviours, without somebody’s help
**04A**	Workers in this institution think that education courses in healthy behaviors are useful
**05A**	The management of this institution works in order to inform about healthy behaviours
**06A**	The management of this institution constantly supports healthy behaviors
**07A**	The management of this institution involves workers in decisions about healthy behaviors
**08A**	Workers in this institution help each other in adopting healthy behaviors
**09A**	Workers in this institution discuss adopting healthy behaviors
**10A**	The management of this institution removes obstacles to healthy behaviors
**Section B**	
**Attitude toward healthy behaviours**	
**01B**	If I implement healthy behaviors, then I prevent health problems
**02B**	If I do not implement healthy behaviors, I feel guilty
**03B**	Implementing healthy behaviors reassure myself
**04B**	When I implement healthy behaviors, I feel better
**05B**	Implementing healthy behaviors calms myself
**06B**	I think I will implement healthy behaviors in the next weeks
**07B**	In the next weeks I will encourage healthy behaviors
**Section C**	
**Implemented healthy behaviours**	
**01C**	I exercise for at least 150 min a week
**02C**	Working with VDU, I take a break every two hours to rest the eyes
**03C**	I eat at least 400 g of fruits and vegetables every day
**04C**	During work I stand up to interrupt the inactive position
**05C**	I drink at least 2 liters of water every day
**06C**	I eat cured meat less than two times a week
